# Polymorphism of the *PPARD* Gene and Dynamic Balance Performance in Han Chinese Children

**DOI:** 10.1186/s41065-019-0092-x

**Published:** 2019-05-23

**Authors:** Yixuan Cao, Qiyue Zhang, Jianhua Chen, Zhiqiang Li, Zhaowei Zhou, Jiawei Shen, Dong Wang, Dun Pan, Zhuo Wang, Dandan Ke, Xiaofei Wang, Dajiang Lu, Ying Zhao, Shulin Cheng, Yongyong Shi

**Affiliations:** 1 Key Laboratory for the Genetics of Developmental and Neuropsychiatric Disorders (Ministry of Education), Bio-X Institutes, Shanghai Jiao Tong University, Shanghai, 200030 China; 2 Shanghai Key Laboratory of Psychotic Disorders, Shanghai Mental Health Center, Shanghai Jiao Tong University School of Medicine, Shanghai, 200030 China; 3 Qingdao University, Metabolic Disease Institute, Qingdao, 266003 China; 4 Department of Human Sports Science, Shanghai University of Sport, Shanghai, 200438 China; 5 Physical Education Department, Shanghai Jiao Tong University, Shanghai, 200240 China; 6 Shanghai Key Laboratory of Sleep Disordered Breathing, Shanghai Jiao Tong University Affiliated Sixth People’s Hospital, Shanghai, 200233 China

**Keywords:** Dynamic balance performance, *PPARD*, PPAR δ, Polymorphisms

## Abstract

**Background:**

Athletic performances are complex traits with heritability of ~66%. Dynamic balance is one of the most important athletic performances, and there has been little studies for it in sports genomics. The candidate *PPARD* gene was reported to be able to affect muscle development for balance predisposition and influence the athletic performance including skiing triumph in the Caucasian population. This study aims to investigate whether the *PPARD* gene is a susceptibility gene for dynamic balance performance in Han Chinese children.

**Results:**

A total 2244 children were recruited and their balance beam performances were measured. Five polymorphisms in the *PPARD* gene were genotyped through the MassARRAY Sequenom platform. Rs2016520 exerted significant association with dynamic balance performance (minor allele C, P = 0.015, P_corrected_ < 0.05) and was affirmed in a meta-analysis by combining previously reported Caucasian cohorts (OR = 1.57, 95% CI = [1.30, 1.91], P < 10 ^-5^) . Another polymorphism, rs2267668, was also significantly associated with dynamic balance performance (minor allele G, P = 0.015, P_corrected_ < 0.05). In the dichotomous study, 321 cases (61% boys and 39% girls) and 370 controls (49% boys and 51% girls) in our samples were selected as representatives, and the thresholds were the mean velocity (0.737 m/s) ± standard deviation (0.264 m/s), in which rs2016520-C and rs2267668-G still remained significant (CI =1.41 [1.11~1.79], P = 0.004, P_corrected_ < 0.016; CI =1.45 [1.14~1.86], P = 0.002, P_corrected_ < 0.016). In different genders, consistent OR direction was observed for each variant.

**Conclusions:**

Our results suggested that the *PPARD* gene is associated with dynamic balance performance of human being, and further studies to reveal its etiology is strongly suggested.

**Electronic supplementary material:**

The online version of this article (10.1186/s41065-019-0092-x) contains supplementary material, which is available to authorized users.

## Background

The heritability of athletic performance is approximately 66%, fluctuating in different sport types [1]. Generally, sports phenotypes are currently divided into explosive power, endurance, strength, flexibility, balance and neuromuscular coordination. Up to now, overall 120 SNPs were found to be associated with athletes’ athletic performance. Among these SNPs, 77 were classified to endurance propensity and 43 to explosive/power propensity [2]. Genetic basis of balance still remains unclear in sports genomics. Sports genomics usually focuses on athletes through case-control studies, and aims to explain the structure and function of genomic regions which might result to better athletic performances [3, 4]. Recently, attention to athletic performance has gradually expanded to lower age groups [5–8]. Notably, basic athletic skills have been developed in early childhood and mature at about 5 to 8 years old [9], therefore, the studies on young children's motor phenotype make sense. Till now, there are far less studies on children than on adults regarding sports genomics. The *ACTN3* gene was found to be associated with children’s explosive power in Han Chinese [10]. *NOS3* and *PPARGC1A* were associated with cardiorespiratory endurance in children with cystic fibrosis [11]. For children, the studied genes are also limited in explaining either explosive/power or endurance propensity, which is the same situation as that in the adults. It is essential to study the genetic basis of other athletic performance which is also of significant importance but remains unclear.

Dynamic balance is the process of maintaining or restoring a stable position while performing a task, and it is vitally important for most human locomotion [12]. The improvement of dynamic balance can enhance a series of athletic performance including vertical jump [13], shuttle-run [14] and downhill skiing [15]. Dynamic balance relies on rapid continuous feedback of vision, vestibular and somatosensory structures to perform coordinated neuromuscular movements [16]. Dynamic balance is one of the physical fitness components that could be health-related or performance-related [17]. In this article, we focused on performance-related components with all the children recruited in healthy status. Sports genomics is promising in explaining individual difference for children's dynamic balance performance.

The transcription factor peroxisome proliferator-activated receptor delta (*PPARD*) gene, which encodes a nuclear hormone receptor PPARδ, is among the hottest studied gene in sports genomics. PPARδ is considered to exert important functions in the process of transcriptional repression and nuclear receptor signaling [18]. It is actively expressed in adipose tissue and skeletal muscles, mainly in type 1 (slow twitch) muscle fibers [19]. Mice studies suggested that PPARδ plays an important role in myelination of the corpus callosum, cell differentiation, epidermal cell proliferation, and lipid metabolism [20–22]. Functional studies suggested that the *PPARD* gene was involved in muscle development and adaptive response to fitness training [23–26]. In mice skeletal muscle, targeted expression of activated PPARδ triggered adaptation from type 2 muscle fibers to type 1 muscle fibers [27]. Type 1 muscle fiber has a beneficial effect on posture and endurance related performance, while type 2 muscle fiber favors strength and power related performance [28]. Large cohort studies have shown that the *PPARD* gene is associated with overall athletic performance in the Polish populations [28] and athletes’ skiing triumph in the Russian populations [29], in which dynamic balance plays a crucial role.. Latest research on the involvement of PPARδ with circadian rhythm inhibitors showed the enhancement of exercise performance in mice via biallelic knock out of PPARδ repressors i.e. *CRY*1 -/- and *CRY2* -/- [30]. However, the biological mechanism of *PPARD* gene is not so clear.

In this study, we hypothesized that the *PPARD* gene might contribute to the individual variance of dynamic balance performance and tried to figure out how it could influence dynamic balance performance. In total, 2244 children were recruited, and their balance beam performances were measured. Five single nucleotide polymorphisms (SNPs) of *PPARD* gene including rs11571504, rs2016520, rs2267668, rs2299869 and rs3798343 were genotyped. Concisely, we conducted a quantitative trait loci (QTL) analysis, a case-control study, and a meta-analysis with one previously published data in the Caucasian population (152 cases and 610 controls) [29] to assess the association between *PPARD* SNPs and dynamic balance performance in Han Chinese children.

## Results

Physical characteristics of all recruited children were summarized in Additional file 1: Table S1. The mean age of the children was 5.36 years old and the average BMI was 16.1 kg/m^2^. The mean velocity walking through the 3-meter long balance beam was 0.737 ± 0.264 m/s. In addition, since a previous study observed correlations between measures of dynamic balance and lower-extremity maximal strength in healthy individuals across the lifespan, in which vertical jump height was one of the measurements to represent maximal strength [31], we also measured vertical jump height of each children for the further analysis. The mean vertical jump height was 17.46 ± 5.46 cm. The balance beam performance and vertical jump height of the children approximately obeyed normal distributions (Additional file 1: Figure S1).

**Fig. 1:**
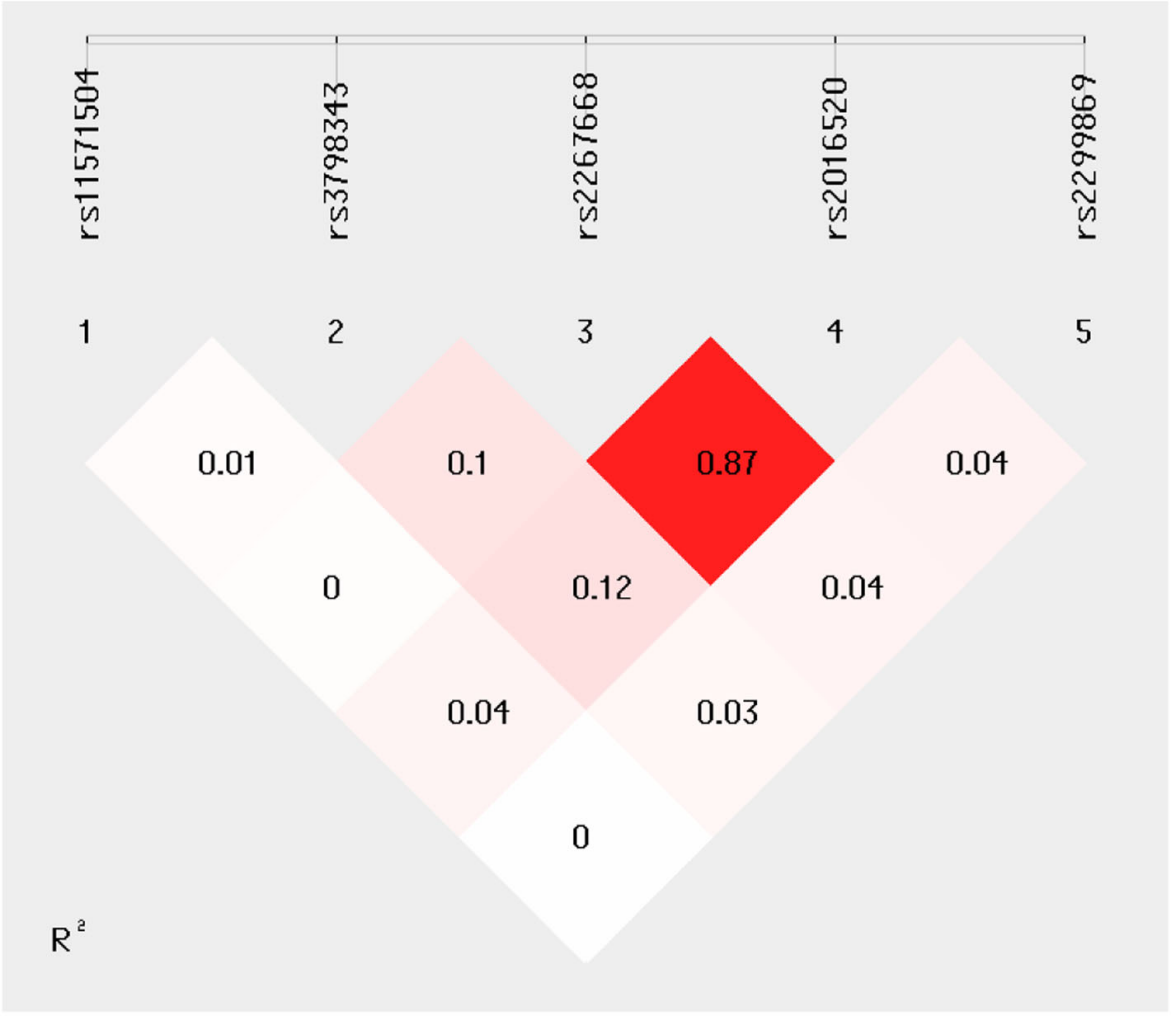
Linkage disequilibrium of five SNPs in the *PPARD* gene

All five SNPs were in Hardy-Weinberg equilibrium (HW_P > 0.05 in Table 1). Fig. 1 illustrated the linkage disequilibrium between rs2267668 and rs2016520. Table 1 shows the quantitative trait loci results of five SNPs in the balance beam performance. Two SNPs (rs2267668 and rs2016520) were significantly associated with dynamic balance performance after multiple testing correction. The SNP rs2016520 showed significance in association with dynamic balance performance (minor allele C, BETA = 0.021 m/s, P = 0.015, P_FDR_ = 0.038), and the significance remained after taking gender, weight, BMI and age as covariates to eliminate their effects respectively (minor allele C, BETA = 0.021 m/s, P_gender_ = 0.02, P_gender_FDR_ = 0.05, P_weight_= 0.013, P_weight_FDR_ = 0.032, P_BMI_ = 0.014, P _BMI-FDR_ = 0.035; BETA = 0.016 m/s, P_age_ = 0.049). Another SNP, rs2267668, was also significantly associated with dynamic balance performance (minor allele G, BETA = 0.023 m/s, P = 0.015, P_FDR_ = 0.038), and P-values maintained significant after correction by gender, weight, BMI and age (minor allele G, BETA = 0.023 m/s, P_gender_ = 0.015, P_gender_FDR_ = 0.05, P_weight_= 0.008, P_weight_FDR_ = 0.032; BETA = 0.022 m/s, P_BMI_ = 0.013, P _BMI-FDR_ = 0.035; BETA = 0.019 m/s, P_age_ = 0.024). The β value in the regression model pointed out the direction of the affecting way. The effect allele rs2016520-C could be a favorable allele favorable for balance beam performance. In a similar way, the β value of rs2267668-G also implied its status as a favorable allele.

**Table 1:** Regression analyzes of five SNPs in the *PPARD* gene for the balance beam performance

SNP	A1	HW_P	Call rate	BETA	P	FDR	BETA^1^	P^1^	FDR^1^	BETA^2^	P^2^	FDR^2^	BETA^3^	P^3^	FDR^3^	BETA^4^	P^4^	FDR^4^
rs11571504	A	0.981	0.951	0.024	0.29	0.363	0.022	0.338	0.423	0.022	0.319	0.362	0.024	0.284	0.355	0.016	0.435	0.444
rs2016520	C	0.998	0.955	0.021	**0.015**	**0.038**	0.021	**0.02**	**0.05**	0.021	**0.013**	**0.032**	0.021	**0.014**	**0.035**	0.016	**0.049**	0.124
rs2267668	G	0.698	0.951	0.023	**0.015**	**0.038**	0.023	**0.015**	**0.05**	0.023	**0.008**	**0.032**	0.022	**0.013**	**0.035**	0.019	**0.024**	0.124
rs2299869	T	0.809	0.951	-0.009	0.427	0.427	-0.008	0.485	0.485	-0.01	0.362	0.362	-0.009	0.421	0.421	-0.01	0.334	0.444
rs3798343	G	0.981	0.951	-0.011	0.211	0.352	-0.011	0.211	0.351	-0.011	0.187	0.312	-0.011	0.202	0.337	-0.006	0.444	0.444

A1, minor allele and the effect allele with which BETA correlates; FDR, Benjamini and Hochberg procedure for controlling the false discovery rate of hypothesis tests, also known as Bonferroni’s correction; BETA^1^, P^1^, FDR^1^: BETA, P, FDR after gender as a covariate to correct; BETA^2^, P^2^, FDR^2^: BETA, P, FDR after weight as a covariate to correct; BETA^3^, P^3^, FDR^3^: BETA, P, FDR after BMI as a covariate to correct; BETA^4^, P^4^, FDR^4^: BETA, P, FDR after age as a covariate to correct; Significance threshold was P < 0.05 and significant P-values were in bold

To preclude interference from lower-extremity maximal strength on the results, independent influence of the *PPARD* polymorphism on dynamic balance and lower-extremity maximal strength was explored, as shown in Table 2 and 3. Significance remained in Table 2 between dynamic balance performance and the *PPARD* polymorphism after corrected by the covariate of vertical jump height (P < 0.05 after corrections). In Table 3, no significance was observed between vertical jump performance and the *PPARD* polymorphism either before or after it was corrected by the covariate of dynamic balance performance (P > 0.05). Both Table 2 and 3 supported that the *PPARD* polymorphism influenced dynamic balance performance rather than lower-extremity maximal strength.

**Table 2:** Vertical jump as a covariate to correct balance beam performance of the 5 SNPs in QTL testing

Phenotype	SNP	A1	BETA	P	FDR	BETA^5^	P^5^	FDR^5^
Balance beam performance	rs11571504	A	0.024	0.29	0.363	0.021	0.196	0.246
	rs2016520	C	0.021	0.015	0.038	0.008	**0.001**	**0.008**
	rs2267668	G	0.023	0.015	0.038	0.008	**0.003**	**0.009**
	rs2299869	T	-0.009	0.427	0.427	0.011	0.47	0.47
	rs3798343	G	-0.011	0.211	0.352	0.008	0.101	0.169

A1, minor allele and the effect allele with which BETA correlates; BETA^5^, P^5^, FDR^5^: BETA, P, FDR after vertical jump as a covariate to correct; Significance threshold was P < 0.05 and significant P-values were in bold

**Table 3:** Balance beam performance as a covariate to correct vertical jump of the 5 SNPs in QTL testing

Phenotype	SNP	A1	BETA	P	FDR	BETA^6^	P^6^	FDR^6^
Vertical jump height	rs11571504	A	-0.09	0.846	0.846	-0.284	0.513	0.855
	rs2016520	C	0.087	0.63	0.63	-0.119	0.479	0.855
	rs2267668	G	0.197	0.301	0.301	-0.017	0.923	0.923
	rs2299869	T	-0.347	0.152	0.152	-0.237	0.294	0.855
	rs3798343	G	-0.144	0.417	0.417	-0.03	0.851	0.923

A1, minor allele and the effect allele with which BETA correlates; BETA^6^, P^6^, FDR^6^: BETA, P, FDR after balance beam performance as a covariate to correct; Significance threshold was P < 0.05 and significant P-values were in bold

We performed gender specific analysis too. Consistent OR directions were observed for each variant in boys and girls groups, which implied the similar predisposition to balance beam performance in different genders. In Table 4, boys’ results showed rs2016520-C (BETA = 0.027 m/s, P = 0.033, P_BMI_ = 0.012, P _BMI-FDR_ = 0.03; BETA = 0.025 m/s, P_weight_ = 0.04, P_age_ = 0.03) and rs2267668-G (BETA = 0.024 m/s, P_BMI_ = 0.013, P_age_ = 0.049) as favorable alleles for dynamic balance performance. In Table 5, girls’ results also displayed the favor of rs2016520-C (BETA = 0.015 m/s, P_weight_= 0.012; BETA = 0.013 m/s, P_BMI_ = 0.012; BETA = 0.014 m/s, P_age_ = 0.047) and rs2267668-G (BETA = 0.023 m/s, P_weight_= 0.012; BETA = 0.021 m/s, P_BMI_ = 0.012) on dynamic balance performance. In comparison with the gender-neutral dataset (Table 1), the significance of rs2016520-C and rs2267668-G slightly decreased in the gender specific dataset (Table 4 and Table 5), the major cause of which was the decline of the sample size resulting from gender split.

**Table 4:** Regression analyzes of five SNPs in the *PPARD* gene for the balance beam performance in 1155 boys

SNP	A1	HW_P	NMISS	BETA	P	FDR	BETA^2^	P^2^	FDR^2^	BETA^3^	P^3^	FDR^3^	BETA^4^	P^4^	FDR^4^
rs11571504	A	0.966	1109	0.046	0.150	0.251	0.043	0.164	0.273	0.046	0.031	0.144	0.031	0.285	0.4
rs3798343	G	0.595	1109	-0.014	0.274	0.342	-0.012	0.327	0.409	-0.014	0.012	0.259	-0.009	0.406	0.406
rs2267668	G	0.971	1108	0.024	0.076	0.191	0.023	0.072	0.18	0.024	**0.013**	0.073	0.024	**0.049**	0.123
rs2016520	C	0.974	1115	0.027	**0.033**	0.163	0.025	**0.04**	0.18	0.027	**0.012**	**0.03**	0.025	**0.03**	0.123
rs2299869	T	0.934	1108	-0.011	0.5314	0.531	-0.013	0.427	0.427	-0.011	0.017	0.53	-0.016	0.32	0.4

A1, minor allele and the effect allele with which BETA correlates; FDR, Benjamini and Hochberg procedure for controlling the false discovery rate of hypothesis tests, also known as Bonferroni’s correction; BETA^2^, P^2^, FDR^2^: BETA, P, FDR after weight as a covariate to correct; BETA^3^, P^3^, FDR^3^: BETA, P, FDR after BMI as a covariate to correct; BETA^4^, P^4^, FDR^4^: BETA, P, FDR after age as a covariate to correct; Significance threshold was P < 0.05 and significant p-values were in bold

**Table 5:** Regression analyzes of five SNPs in the *PPARD* gene for the balance beam performance in 1089 girls

SNP	A1	HW_P	NMISS	BETA	P	FDR	BETA^2^	P^2^	FDR^2^	BETA^3^	P^3^	FDR^3^	BETA^4^	P^4^	FDR^4^
rs11571504	A	0.999	1026	-0.01	0.762	0.762	-0.01	0.032	0.743	-0.01	0.032	0.747	-0.009	0.657	0.742
rs3798343	G	0.708	1026	-0.008	0.524	0.762	-0.01	0.011	0.381	-0.007	0.011	0.525	-0.002	0.745	0.846
rs2267668	G	0.604	1026	0.021	0.096	0.478	0.023	**0.012**	0.056	0.021	**0.012**	0.088	0.013	0.056	0.343
rs2016520	C	0.951	1028	0.013	0.287	0.718	0.015	**0.012**	0.204	0.013	**0.012**	0.28	0.014	**0.047**	0.21
rs2299869	T	0.884	1025.000	-0.006	0.726	0.762	-0.005	0.015	0.722	-0.005	0.015	0.718	-0.003	0.746	0.846

A1, minor allele and the effect allele with which BETA correlates; FDR, Benjamini and Hochberg procedure for controlling the false discovery rate of hypothesis tests, also known as Bonferroni’s correction; BETA^2^, P^2^, FDR^2^: BETA, P, FDR after weight as a covariate to correct; BETA^3^, P^3^, FDR^3^: BETA, P, FDR after BMI as a covariate to correct; BETA^4^, P^4^, FDR^4^: BETA, P, FDR after age as a covariate to correct; Significance threshold was P < 0.05 and significant p-values were in bold

To further verify the association between the *PPARD* gene and balance beam performance, the mean velocity (0.737 m/s) and ± standard deviation (0.264 m/s) were used as the thresholds to define the case and the control group. Concisely, 321 children(61% boys and 39% girls)were picked up as the case group for their velocity above the mean value plus standard deviation (average velocity ± standard deviation: 1.16 ± 0.14 m/s) and 370 children (49% boys and 51% girls) were selected as the control group for their velocity below the mean value minus standard deviation (average velocity ± standard deviation: 0.37 ± 0.08 m/s). Table 6 and Table 7 demonstrates the case-control analysis results. In 321 cases and 370 controls, the two SNPs (rs2016520, rs2267668) remained significantly associated with the phenotype of balance beam performance after multiple corrections, i.e. the genetic predisposition of rs2016520-C (CI =1.41 [1.11~1.79], P = 0.004, P_FDR_ = 0.012 in Table 6, P _covariate corrections_ < 0.05 in Table 7) and rs2267668-G (CI =1.45 [1.14~1.86], P = 0.002, P _FDR_ = 0.012 in Table 6, P _covariate corrections_ < 0.05 in Table 7) was consistent with the effect in the previous QTL analysis.

**Table 6:** Case-control study on balance beam performance

SNP	Call rate	OR 95% CI	Genotype frequency			P	FDR	Allele frequency		P	FDR
rs11571504	0.982	1.33 [0.7~2.55]	AA	AT	TT	0.265	0.389	A	T	0.379	0.379
Case			0(0.000)	20(0.063)	298(0.937)			20(0.031)	616(0.969)		
Control			1(0.003)	16(0.042)	362(0.955)			18(0.024)	740(0.976)		
rs3798343	0.982	0.85 [0.68~1.07]	CC	CG	GG	0.212	0.347	C	G	0.159	0.265
Case			158(0.497)	135(0.425)	25(0.079)			451(0.709)	185(0.291)		
Control			177(0.467)	157(0.414)	45(0.119)			511(0.674)	247(0.326)		
rs2267668	0.982	1.45 [1.14~1.86]	AA	AG	GG	**0.005**	**0.013**	A	G	**0.002**	**0.012**
Case			161(0.506)	133(0.418)	24(0.075)			455(0.715)	181(0.285)		
Control			238(0.628)	119(0.314)	22(0.058)			595(0.785)	163(0.215)		
rs2016520	0.973	1.41 [1.11~1.79]	CC	CT	TT	**0.004**	**0.013**	C	T	**0.004**	**0.012**
Case			26(0.081)	143(0.445)	152(0.474)			195(0.304)	447(0.696)		
Control			26(0.070)	123(0.332)	221(0.597)			175(0.236)	565(0.764)		
rs2299869	0.982	0.86 [0.62~1.19]	CC	CT	TT	0.541	0.509	C	T	0.35	0.379
Case			251(0.789)	63(0.198)	4(0.013)			565(0.888)	71(0.112)		
Control			291(0.768)	79(0.208)	9(0.024)			661(0.872)	97(0.128)		

FDR, Benjamini and Hochberg procedure for controlling the false discovery rate of hypothesis tests, also known as Bonferroni’s correction; Significance threshold was P < 0.05 and significant p-values were in bold

**Table 7:** Gender, weight, BMI and age as covariates in case-control study of balance beam performance

SNP	A1	HW_P	OR 95% CI^1^	P^1^	FDR^1^	OR 95% CI ^2^	P^2^	FDR^2^	OR 95% CI^3^	P^3^	FDR^3^	OR 95% CI^4^	P^4^	FDR^4^
rs11571504	A	0.999	1.37 [0.72-2.6]	0.338	0.422	1.31[0.69~2.51]	0.409	0.409	1.3[0.69~2.46]	0.423	0.423	1.17 [0.48~2.85]	0.736	0.736
rs3798343	G	0.904	1.19 [0.95-1.49]	0.139	0.233	0.84[0.67~1.06]	0.149	0.248	0.84[0.67~1.05]	0.133	0.221	0.85 [0.64~1.13]	0.268	0.335
rs2267668	G	0.856	1.44 [1.13-1.84]	**0.004**	**0.016**	1.47[1.14~1.88]	**0.002**	**0.014**	1.461[1.14~1.87]	**0.002**	**0.01**	1.5 [1.1~2.05]	**0.01**	0.051
rs2016520	C	0.935	1.4 [1.1-1.78]	**0.006**	**0.016**	1.41[1.1~1.8]	**0.006**	**0.016**	1.42 [1.12~1.80]	**0.004**	**0.01**	1.37 [1.01~1.86]	**0.041**	0.103
rs2299869	T	0.775	0.89 [0.64-1.22]	0.461	0.461	0.84[0.6~1.16]	0.285	0.356	0.86[0.63~1.19]	0.374	0.423	0.77 [0.52~1.15]	0.195	0.326

A1, minor allele and the effect allele with which BETA correlates; FDR, Benjamini and Hochberg procedure for controlling the false discovery rate of hypothesis tests, also known as Bonferroni’s correction; BETA^1^, P^1^, FDR^1^: BETA, P, FDR after gender as a covariate to correct; BETA^2^, P^2^, FDR^2^: BETA, P, FDR after weight as a covariate to correct; BETA^3^, P^3^, FDR^3^: BETA, P, FDR after BMI as a covariate to correct; BETA^4^, P^4^, FDR^4^: BETA, P, FDR after age as a covariate to correct; Significance threshold was P < 0.05 and significant p-values were in bold

**Fig. 2:**
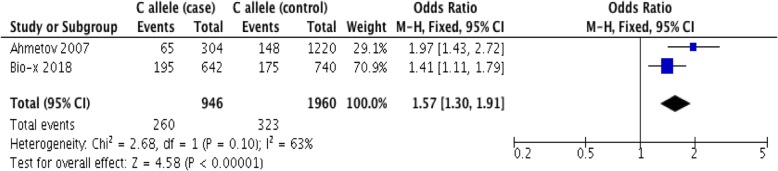
meta-analysis information of rs2016520’s effect on dynamic balance related performance

Additionally, we performed meta-analysis by integrating our case- control data with similar data from one previously reported study. A genomic study on the association of the *PPARD* polymorphism and skiing triumph among the Russian population was selected in our meta-analysis. Skiing is considered to be a sort of sport largely depended on dynamic balance [32–34]. In real skiing situation, there are movements like turning, vacating and diverting in the air, which requires dynamic balance to restore a stable position while performing the skiing task, therefore, dynamic balance is one of the main factors that restricts skiing triumph [34]. This Russian study recruited 152 athletes with competitive standards in skiing (including alpine skiing, ski jumping, cross-country skiing 5-10 km and 15–50 km) as cases and 610 non-athletes as controls, and then conducted genotyping to do association study [29]. Similar research method as ours were seen in their study, therefore we selected this study to carry out the meta-analysis. With the inaccessibility of detailed individual information in the Russian study, it was unable to conduct covariate corrections. The two populations were not significantly heterogeneous (heterogeneity P-value = 0.1, I^2^ = 63% in Fig. 2), so that the fixed-effect model was used to estimate the overall effect of rs2016520-C on dynamic balance performance [35]. Although no significant heterogeneity was seen in the two populations, the influence of differences in age, weight, training status and other factors on the analyses and results was not completely excluded. Fig. 2 shows that the favor of dynamic balance was linked to rs2016520-C allele based on the fixed-effect model(OR = 1.57, 95% CI = [1.30, 1.91], P < 10 ^-5^). In different races, the P-values remained significant and the OR values were in the same direction. Chinese children and European athletes share consistent genetic predisposition on dynamic balance related performance. People owning rs2016520-C tends to have better dynamic balance related performance.

## Discussion

Our findings suggested that common SNPs in the *PPARD* gene could play an important role in Children’s dynamic balance performance. In general, the rs2016520-C allele could promote dynamic balance performance concluded from QTL analysis (BETA > 0, P < 0.05 after correction as shown in Table 1), case-control study (CI > 1, P < 0.05 after correction as shown in Table 6 and 7) and meta-analysis confirmation with the Caucasian population (OR = 1.57, 95% CI = [1.30, 1.91], P < 10 ^-5^ in Fig. 2). Consistent BETA directions were observed for each variant in different genders, implying the similar predisposition to balance beam performance. In both genders, rs2016520-C favors better dynamic balance performance than rs2016520-T (BETA > 0 in Table 4 and 5).Although significant age difference were observed in other studies [31], significance remained in this study after taking age as a covariate (P < 0.05 in Table 1, 4, 5 and 7), which might be due to the elementary mature of basic motor skills in 5-8 years old children [9]. The previously reported associations between measures of dynamic balance and lower-extremity maximal strength [31] did not interfere the effect of the *PPARD* polymorphism on the dynamic balance performance in our study (P < 0.05 after correction in Table 2; P > 0.05 in Table 3). Studies showed that the function of neurophysiological mechanisms during dynamic balance control and maximal strength production is task-specific, although the activation of corticospinal pathways are similar [36, 37]. Furthermore, studies of spinal and corticospinal excitability showed different activation patterns in task-specific performance. For example, during the execution of strength-related isometric ankle dorsiflexion and plantarflexion, short-latency facilitation induced by transcranial magnetic stimulation was observed [38], yet it was not seen during the performance of a balance-related perturbed stance task [39].

Rs2016520, located in the 5’ untranslated region of exon 4, is the best clinically studied polymorphism in the *PPARD* gene. The 39% higher transcriptional activity of the *PPARD* promotor was observed within the mutant rs2016520-C owners than the common T allele ones [40]. Given that the rs2016520-T mutate into the rare C allele, the bilateral nucleotides become consensus sequence for Sp-1 binding, which might increase the affinity of Sp-1 transcriptional factor and explain the up-regulation of *PPARD* expression for rs2016520-C owners [40]. Moreover, studies showed that activation of PPARδ triggered adaptation in the skeletal muscle from type 2 muscle fibers (strength and power related) to type 1 muscle fibers (postural and endurance related), enabling the mutant mice to run a longer distance in comparison with the wildtype [23, 27]. Large cohort studies in human also showed consistency in the *PPARD* gene regarding association with elite athlete’s performances in the European populations [28, 29]. In particular, the rs2016520-C allele was associated with the triumph of skiing in the Russian population [29], in which dynamic balance could play a rather crucial role. In this study, we replicated a significant association of rs2016520-C with dynamic balance performance in the Han Chinese children. After multiple corrections and further confirmation among the different populations, our results suggest the rs2016520-C as a favorable allele for dynamic balance performance.

rs2267668-G was significantly associated with dynamic balance performance concluded from QTL analysis (BETA > 0, P < 0.05 after correction in Table 1) and case-control analysis (CI > 1, P < 0.05 after correction in Table 6 and 7). In the boys’ or girls’ dataset, BETA directions were observed consistent with gender-neutral ones ( BETA > 0 for rs2267668-G, shown in Table 1, 4 and 5), although the significance decreased due to the split of the sample size. In both genders, rs2267668-G favors better dynamic balance performance. Previous studies showed that *PPARD* rs2267668 A/G SNP had impacts on the improvement of mitochondrial function and aerobic physical fitness [41, 42]. A blunted increase in individual anaerobic threshold (i.e. worse aerobic physical fitness) was observed in rs2267668-G, which suggested the less effectiveness of aerobic exercise training, while rs2267668-G of cultured human myotubes displayed low skeletal muscle mitochondrial function [41]. In a physical and dietary lifestyle intervention, the minor G allele of rs2267668 was associated with less reduction in nonvisceral adipose tissue mass as well as blunted increase in relative muscle volume of the leg [42]. The G allele of rs2267668 showed better dynamic balance performance in Chinese children in our study. The influence effect of the rs2267668 site still needs further investigation from experiments. The marginal positive result of the correlation between rs2267668 and dynamic balance performance might be caused by the linkage disequilibrium with the 5'UTR functional rs2016520.

## Conclusion

In summary, we found the significant association of one 5’UTR variant (rs2016520-C) and one intron variant (rs2267668-G) in the PPARD gene with the balance beam performance. Our results suggest that the *PPARD* gene is associated with dynamic balance performance of children, and further studies to reveal its etiology is strongly suggested.

## Methods

### Recruited volunteers’ dataset

With the purpose of the study clearly explained to the parents, children, and all informed consent accessible to the children’s guardians, our study was scrutinized and approved by the local Ethical Committee of Human Genetics. The research was conducted in accordance with institutional requirements and the Declaration of Helsinki Principles. Overall 2244 unrelated healthy Chinese children (1155 boys and 1089 girls; average age ± standard deviation: 5.36 ± 1.03 years old) were recruited. All the children were of Han Chinese origin and lived in Shanghai. Oral epithelial cells were collected through oral swabs in identified tubes for follow-up experiments.

### Phenotypes measurement

All the tests of balance beam performance and vertical jump height were conducted and recorded by the Shanghai University of Sports based on the standard manual for Chinese national physique determination (child part) [43]. We made efforts to ensure the children to fully understand the process of measurements and reflect their true levels. The performance of walking through the balance beam was to evaluate children's dynamic balance performance. The balance beam was 3 meters long, 10 centimeters wide and 30 centimeters high. A 20 centimeters long, 20 centimeters wide, and 30 centimeters high board were added for step to each end of the balance beam as shown in Additional file 1: Figure S2. During the test, the children were guided to stood on the board and faced the balance beam with arms lifted horizontally. When ‘start’ was heard, the children proceeded as fast as possible. Walking through time was recorded and then the average velocity was calculated for further research. As for the vertical jump test, the Vertec apparatus was used. It was of steel frame construction with horizontal vanes which were rotated out of the way by the hand to indicate the height reached. We firstly recorded the standing height of each child with one arm fully extended upward, then instructed them to jump upwards vigorously and touch the highest possible vane. The vertical jump height was the difference between the maximal jump height and standing height. The score was recorded in centimeters. Each test allowed the children to perform three times to pick out the best score for the analysis. The same equipment under the same condition was used in each time to eliminate systematical errors.

### SNPs selection and genotyping

The *PPARD* gene is located on chromosome 6p21.2-p21.1 and it spans about 92.62 kb of DNA and contains several SNPs. We downloaded the *PPARD* gene information of the Southern Han Chinese population from the 1000 Genomes website and selected 5 common tag SNPs (rs3798343, rs2299869, rs2267668, rs11571504, and rs2016520) as shown in Additional file 1: Figure S3 and Additional file 1: Table S2 through the Haploview 4.2 Software (Ensembl GRCh37 2018) [44]. To be exact, the downloaded marker information and linkage pedigree format were firstly uploaded to the Haploview 4.2 Software. Then, major thresholds were set as listed below: HW-P value cutoff at 0.05, minimal genotype percentage at 95%, maximal Mendel errors at 1, minor allele frequency (MAF) at 0.05, r^2^ threshold at 0.8 [45]. For the linkage format, pairwise comparisons of markers over 500 kb apart were set to be ignored. Afterwards, ‘Pairwise tagging only’ model was selected and the five tag SNPs were then given by the software. TIANamp Swab DNA Kit (TIANGEN) was used to extract genomic DNA from oral epithelial cells. The genomic DNA was obtained from participants’ epithelial mouth cells using high yield oral swab genomic DNA extraction kit. 2 μl DNA solution of each child was used for genotyping. All SNPs were genotyped on the Sequenom Massarray platform in Bio-x institute. SNPs were all genotyped by a call rate ≥ 95%, which suggested the failure rate of genotyping below 5%. The genotyping rate of each SNP in each studied group was listed in the relevant table. The process of Sequenom Massarray SNP genotyping involved polymerase chain reaction (PCR), Shrimp alkaline phosphatase (SAP) reaction, single base extension, resin cleanup and detection on mass spectrometry [46].

### Statistical analysis

We used SHEsisPlus platform [47–49] to carry out statistical analysis, including Hardy–Weinberg equilibrium (HWE), linkage disequilibrium (LD), quantitative trait loci(QTL)and case-control analysis for 5 SNPs in the *PPARD* gene. QTL analyses were firstly carried out to find out the genotypes that might be related to the quantitative trait. The multiple-testing procedure was used to eliminate the duplicate effects of 5 SNPs to control the false discovery rate [50]. The covariate of gender, weight, BMI, age and vertical jump height were further used in the regression model to eliminate their effect on the balance beam performance based on SHEsisPlus [47–49]. After taking these factors as covariates to eliminate their effects on dynamic balance performance, the sports genomics studies could be more meaningfully interpreted. The statistical significance was set at the threshold P < 0.05. The OR value in the case-control analysis indicates the influence direction and intensity of the *PPARD* loci on the balance trait. CI values of the Han Chinese children are used to estimate the range of the children's overall parameters. In addition, we searched related literature to conduct meta-analysis, which was carried out on RevMan5.3 software under Cochran guidelines [33]. The Z-test was used to detect significance and the heterogeneity Q test was used to assess whether effects differ among different populations. In this test, the heterogeneity P > 0.05 (Fig. 2) suggests that the two populations are a homogeneous collection. Therefore, the fixed effect Mantel-Haenszel model was used.

## Additional file

Additional file 1: **Table S1.** Physical characteristics of all recruited children. **Table S2.** Genetic information of five markers. **Figure S1.** Distribution of the balance beam and vertical jump performance in the Han Chinese children. **Figure S2.** Diagram of balance beam performance. **Figure S3.** Location of the five single nucleotide polymorphisms (SNPs) in the PPARD gene. (DOCX 365 kb)

## Abbreviations

CEU: Utah residents with Northern and Western Europe
CHR: Chromosome
CHS: Han Chinese in South
FDR: Benjamini and Hochberg procedure for controlling the false discovery rate of hypothesis tests
HWE: Hardy- Weinberg equilibrium
MAF: minor allele frequency
OR: Odds ratio
*PPARD*/ PPARδ: peroxisome proliferator-activated receptors delta
SD: Standard deviation
SNP: Single nucleotide polymorphism
