# Supplementary material for: Polymorphism of the PPARD Gene and Dynamic Balance Performance in Han Chinese Children
Source: Hereditas. 2019 May 23;156:15. doi: 10.1186/s41065-019-0092-x (PMC6533762; doi:10.1186/s41065-019-0092-x)
Supplement: Supplementary file 1 — Table S1. Physical characteristics of all recruited children. Table S2. Genetic information of five markers. Figure S1. Distribution of the balance beam and vertical jump performance in the Han Chinese children. Figure S2. Diagram of balance beam performance. Figure S3. Location of the five single nucleotide polymorphisms (SNPs) in the PPARD gene. (DOCX 365 kb) [file 41065_2019_92_MOESM1_ESM.docx]

**Table S1** Physical characteristics of all recruited children

| gender |  | age（yr） | BMI (kg/m^2^) | number |
| --- | --- | --- | --- | --- |
| boy | average | 5.37 | 16.37 | 1089 |
|  | (SD) | (±1.04) | (±2.8) |  |
| girl | average | 5.34 | 15.82 | 1155 |
|  | (SD) | (±1.03) | (±2.52) |  |
| sum | average | 5.36 | 16.1 | 2244 |
|  | (SD) | (±1.03) | (±2.68) |  |

SD, Standard deviation; yr, years old

**Table S2** Genetic information of five markers

| Marker | Position1 | Function^2^ | Polymorphism^1^ | Minor allele^1^ | MAF1 CEU | MAF1 CHS |
| --- | --- | --- | --- | --- | --- | --- |
| rs3798343 | Chr6: 35,357,693 | intron variant | C/G | G | G=0.02 | G=0.286 |
| rs2299869 | Chr6: 35,383,432 | intron variant | C/T | T | T=0 | T=0.176 |
| rs2267668 | Chr6: 35,377,922 | intron variant | A/G | G | G=0.182 | G=0.281 |
| rs11571504 | Chr6: 35,310,749 | intron variant | A/T | A | A=0.066 | A=0.019 |
| rs2016520 | Chr6: 35,378,778 | 5'UTR | C/T | C | C=0.212 | C=0.3 |

1, on the assembly GRCh37.p13 in 1000 Genomes website; 2, collect from NCBI database of assembly GRCh37.p13; MAF, minor allele frequency; CEU, Utah residents with Northern and Western Europe; CHS, Han Chinese in South

**Fig. S1** distribution of the balance beam and vertical jump performance in the Han Chinese children


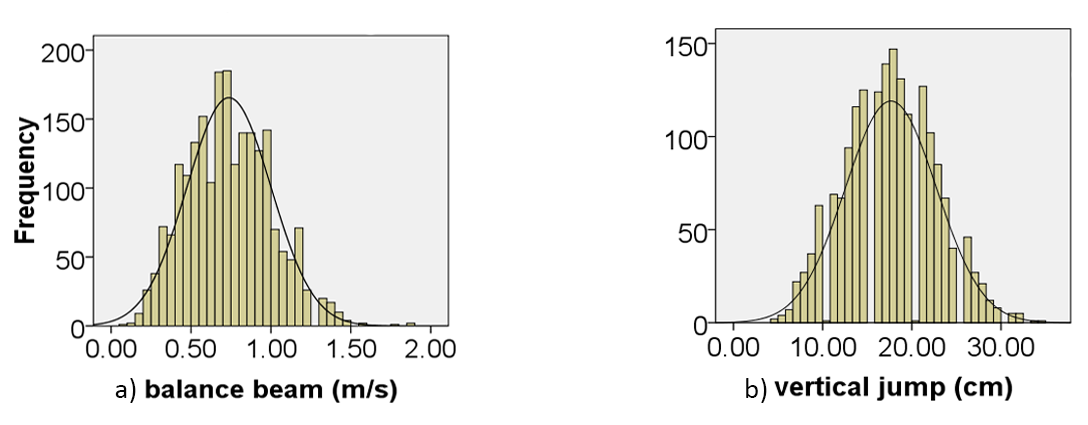

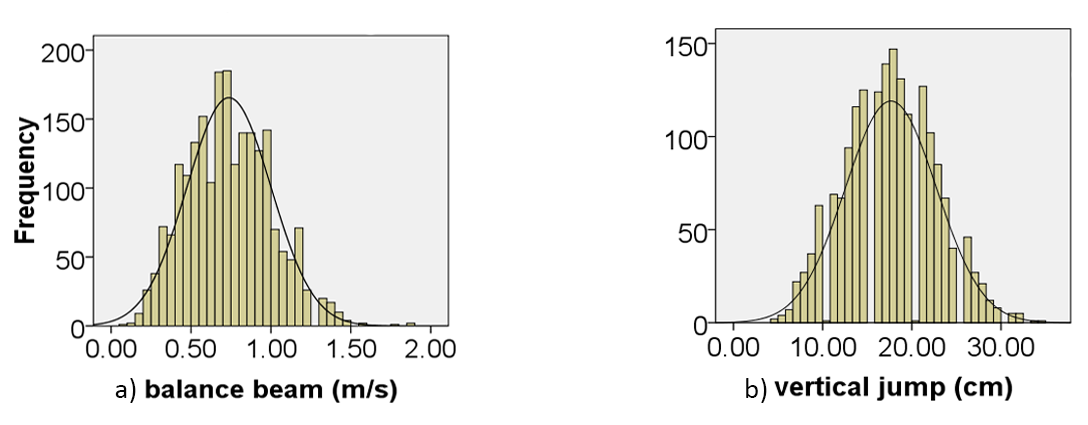


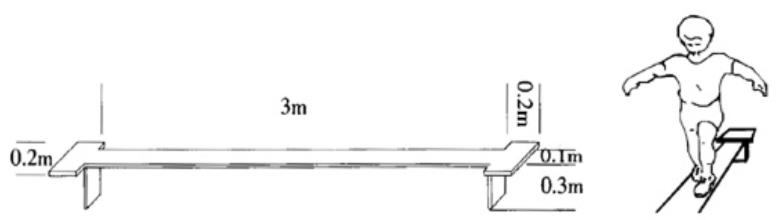


**Fig. S2** Diagram of balance beam performance


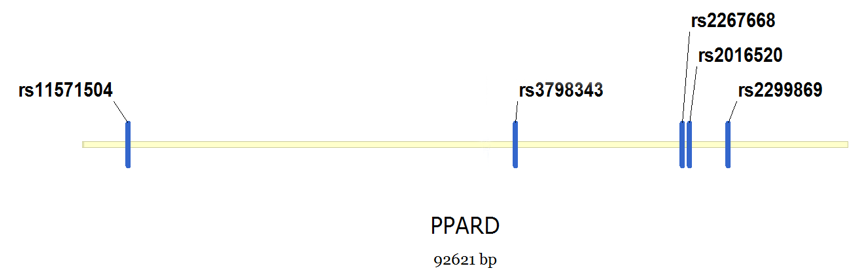


**Fig. S3** Location of the five single nucleotide polymorphisms (SNPs) in the PPARD gene
